# The expression and significance of proto-oncogene c-fos in viral myocarditis

**DOI:** 10.1186/1743-422X-7-285

**Published:** 2010-10-27

**Authors:** Song Zhang, Ben He, Steven Goldstein, Junbo Ge, Zuyue Wang, George Ruiz

**Affiliations:** 1 Department of Cardiovascular Diseases, Eastern District of Renji Hospital, Shanghai Jiaotong University, 1630 Dongfang Road, Shanghai, 200127, China; 2 Department of Cardiovascular Diseases, People's Hospital of Zhejiang Province,158 Shangtang Road, Hangzhou, Zhejiang, 310014, China; 3 Department of Cardiovascular Diseases, Zhongshan Hospital, Fudan University, 180 fenglin Road, Shanghai, 200032, China; 4 Department of Cardiology, Washington Hospital Center, 110 Irving Street, Washington, DC, 20010, USA

## Abstract

**Background:**

c-fos may play a role in the pathogenesis of some diseases. The expression and function of c-fos in viral myocarditis (VMC) have not yet been reported. To study the change and significance of proto-oncogene c-fos in VMC is the objective of this experiment.

**Methods:**

An animal model of VMC was established via coxsackie virus B_3 _inoculation. VMC mice were then treated with a c-fos monoclonal antibody and isoproterenol and the protein and mRNA expression of c-fos were studied via immunohistochemical analysis and *in situ *hybridization. Results were simultaneously analyzed for the significance of c-fos expression in mice with VMC.

**Results:**

Myocardial necrosis and cell infiltration decreased after treatment with c-fos monoclonal antibody compared to control mice, while myocardial necrosis and cell infiltration were increased after treatment with isoproterenol. Positive cardiomyocytes with c-Fos expression increased at 3, 5, 7, 9, and 15 days after virus inoculation in VMC mice compared to control mice, while returning to almost normal levels at 35 days. The expression level of c-fos mRNA at 3 and 7 days after virus inoculation in VMC mice was also higher than that of control mice.

**Conclusions:**

c-fos expression in the cardiomyocytes of VMC mice is significantly increased, c-fos plays an important role in myocardial lesions. The apparent increase in expression of c-fos is likely to be involved in the pathogenesis of VMC.

## Background

The proto-oncogene c-fos participates in a variety of physiological process including cell growth, differentiation, transformation, signal transduction, and plasticity of the nervous system [1]. The expression of c-fos is known to be increased in particular diseases and pathophysiological processes, indicating that it may play a role in the pathogenesis of some diseases. The expression and function of c-fos in viral myocarditis (VMC) have not yet been reported. Therefore, our experiments were focused on the study of the expression of c-fos in VMC by ways of immunohistochemical analysis and *in situ *hybridization. Simultaneously, we investigated the significance of c-fos in VMC via medicine treatment with c-fos monoclonal antibody or isoproterenol.

## Materials and Methods

### Animals

BALB/c mice, male, 4-6 weeks old, 16-20 grams.

### Main reagents

c-fos monoclonal antibody, isoproterenol, normal goat serum, rabbit anti-c-fos oncogene protein, Biotinylated goat anti rabbit IgG, Streptavidin Biotin-peroxidase Complex (SABC), antigen restoration solution, pepsin, c-fos oligonucleotide probe, Occlusive solution, and rabbit anti digoxin were purchased from Boster Biological Technology Ltd.(Wuhan, China), Sigma Chemical Co. (Sigma, St.Louis, MO) and Biocompare Co.(South San Francisco, CA).

### Establishing of animal model (VMC)

130 mice were divided into two groups: the experimental group (120 mice) and the control group (10 mice). Each mouse of the experimental group was inoculated with coxsackie virus B_3 _(CVB_3_), while control mice were inoculated with MEM 0.1 mL Eagle's solution. Experimental mice were sacrificed at day (D) 3, 5, 7, 9, 15, and 35 after inoculation (Groups D_3_, D_5_, D_7_, D_9_, D_15, _and D_35_). 120 mice were included in the experiment group, but some mice died, some mice lost, some mice bit each other lead to death and also because of some other reasons, we only got 60 specimens at last. Dead mice number of every sub-group: 5 in GroupD_3_, 6 in Group D_5_, 8 in Group D_7_, 7 in Group D_9_, 8 in Group D_15 _and 7 in Group D_35_.

### Medicine treatment

Another one hundred and twenty mice were divided into three groups of 40 mice each (GroupE_1, _E_2, _and E_3_). Each group of mice was inoculated with 0.1 mL of coxsackie virus B_3 _(CVB_3_). Each mice of Group E_1 _was then inoculated with 5 μg of c-fos monoclonal antibody via intraperitoneal injection every day for 3 days. Group E_2 _mice were inoculated with 1 μg of isoproterenol every day for three days. Group E_3 _mice were inoculated with 0.1 mL of normal saline for three days. Each group was divided into two subgroups (20 mice/subgroup), in which one subgroup was sacrificed on day 7, and the other was sacrificed at day 15.

### Specimen collection

Serum was isolated from blood samples and refrigerated for further use. Each heart specimen was divided into two portions, in which one portion was fixed with 10% methanol. Paraffin-embedded tissue samples were cut into 5 μm sections and stained with hematoxyline/eosin according to standard procedures and observed under a light microscope (Olympus). The remaining portion of the sample was preserved with glutaric dialdehyde to be used for electron microscopy.

### Immunohistochemical and in situ analysis of the c-fos oncogone

Heart specimens were fixed in 10% methanol for 24 hours and paraffin-embedded tissue samples were cut into 5 μm sections. 5 sections were used in every example, sections were mounted on 3-aminopropyltriethoxysilane (APES) treated slides followed by incubation at 56°C for 1-2 hours, followed by 37°C incubation for 3 days.

Immunohistochemical analysis of c-Fos oncogene protein: After standard deparaffination and rehydration, specimens were exposed to xylol for 10 minutes, 100% alcohol for 5 minutes, 96% alcohol for 5 minutes, and 70% alcohol for 3 minutes. Endogenous peroxidase activity was quenched by exposure to 3% hydrogen peroxide for 10 minutes. The antigen was restored by citrate-buffered (pH 6.0). Normal goat serum was added for 10 minutes at room temperature, c-Fos antibody was added at 37°C for 1.5 hours followed by washing with phosphate-buffered saline (PBS). Biotinylated goat anti-rabbit IgG was added for 20 minutes at 37°C followed by a PBS wash. Streptavidin Biotin-peroxidase Complex (SABC) was added for 20 minutes at 37°C and then rinsed with PBS. The color was then developed with diaminobenzidine (DAB) at room temperature, and rinsed with distilled water after the reactive time was controlled under the light microscope. Sections were restained with hematoxylin, and incubated at 37°C and sealed with neutral gum. Slides were observed under the light microscope.

*In situ *hybridization of the c-fos oncogene: Formalin-fixed paraffin-embedded heart specimens were deparaffinized with xylene and rehydrated with graded ethanol. The endogenous peroxidase activity was quenched by exposure to 3% hydrogen peroxide for 10 minutes. Samples were then incubated with pepsin (diluted with 3% citric acid) at 37°C for 20 minutes and rinsed with 0.5 M PBS and distilled water.

The Digoxin-labeled probe was added to the sections and sections were then covered with coverslips and incubated overnight at 37°C. After the coverslips were disclosed, the sections were rinsed with 2×SSC (17.6 g sodium chloride and 8.8 g sodium citrate in 1000 mL of distilled water), and 0.2 × SSC (1:10 dilution from 2×SSC). Rabbit anti-Digoxin was added to the sections for 60 minutes at 37°C, then rinsed with 0.5 M PBS. Biotinylated goat anti-rabbit IgG was added for 30 minutes at 37°C, then rinsed with 0.5 M PBS. SABC was added for 30 minutes at 37°C, then rinsed with 0.5 M PBS. Sections were colored with DAB, and the reactive time was controlled under the light microscope. Sections were restained with hematoxylin and sealed with neutral gum and was observed under the light microscope.

### Determination of results

A blue cell nucleus indicated a normal cardiomyocyte, while a brown-yellow nucleus indicated positive expression in the cardiomyocyte of the c-Fos oncogene protein. Brown-yellow particles in the cytoplasm indicated positive expression of c-fos oncogene mRNA. The number of positive cell nucleus (or cytoplasm) of five high-power fields were calculated under light microscope, average value was calculated.

### Histopathological Examination

One section of the heart specimen was fixed in 10% formalin, embedded in paraffin, stained with hematoxylin and eosin, and then observed by microscopy at 200 × magnification. According to the myocardial lesions, including cell necrosis and cellular infiltration, each specimen was given a score by two observers who were unaware of the treatment group. Histopathological scores were evaluated as follows: 0, no lesions; 1, lesions involving <25% of the myocardium; 2, lesions involving 25% to 50% of the myocardium; 3, lesions involving 50% to 75% of the myocardium; and 4, lesions involving >75% of the myocardium.

### Statistics

All data were expressed as mean ± standard deviation (SD). A *t-*test or variance analysis was used to compare data between groups. A level of *p *< 0.05 was considered to be statistically significant.

## Results

### Establishing an animal model of VMC(Evidence of VMC)

Signs of VMC were apparent in the experimental groups at 3 days after virus inoculation including coat ruffling, weakness, and irritability. On day 3, a few scattered small foci of myocyte necrosis were noted. Myocardial necrosis and cell infiltration were extensive on day 7, with necrotic areas appearing more prominent. There were many lymphocytes and macrophages in and around the necrotic foci. Infiltration of the inflammatory cells and necrotic areas were decreased, and necrotic myocardium gradually changed to fibrosis and calcification on day 15, at which time fibrosis was noted in the interstitium. There were no necrotic lesions or signs of cell infiltration in the hearts of uninfected control mice.

### Expression of c-Fos oncogene protein in VMC mice

**Figure 1:**
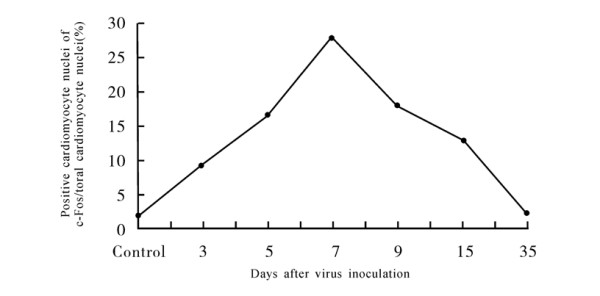
**The proportion change of positive cardiomyocyte nucleus of c-Fos protein in VMC mice**.

**Figure 2:**
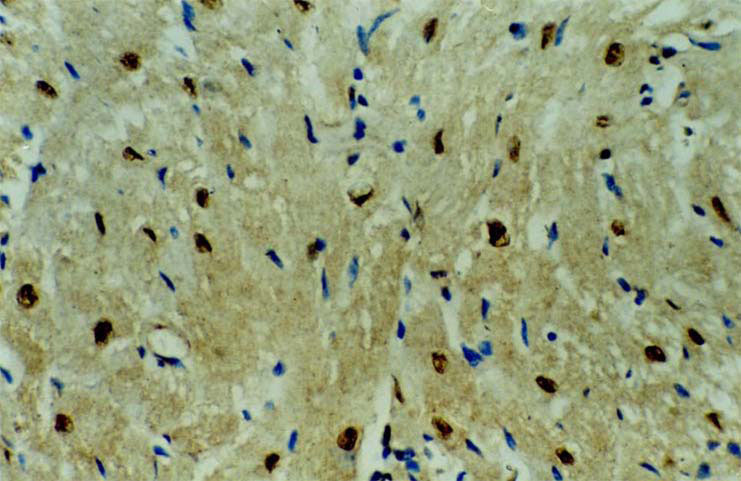
**The expression of c-Fos protein in cardiomyocytes of VMC mice at 9 days after virus inoculation (400×)**.

A few c-Fos oncogene protein positive cardiomyocyte nuclei were seen in mice of the control group. Positive expression of c-Fos protein increased significantly at 3 days after virus inoculation in VMC mice. The proportion of positive cardiomyocyte nucleus and total cardiomyocyte nucleus also increased apparently with the advance of the disease. The peak level was at 7-9 days after virus inoculation (Table 1, Figures 1 and 2). Positive c-Fos protein expression in cardiomyocyte nuclei was almost normal at 35 days after virus inoculation.

**Table 1:** The expression change of c-Fos oncogene protein in VMC mice

Group	Number	PCN/HPF	PCN/TCN(%)
D_3_	7	43.86 ± 14.18^Δ^	9.52 ± 2.80^Δ^
D_5_	8	66.63 ± 21.71^Δ^	16.73 ± 5.76^Δ^
D_7_	8	109.79 ± 29.25^Δ^	27.92 ± 7.87^Δ^
D_9_	8	75.19 ± 20.67^Δ^	18.26 ± 4.71^Δ^
D_15_	10	56.64 ± 21.06^Δ^	13.62 ± 5.08^Δ^
D_35_	7	9.37 ± 4.07	2.37 ± 1.20
Control	10	8.25 ± 2.44	2.03 ± 0.60

PCN: Positive cardiomyocyte nucleus; TCN: Total cardiomyocyte nucleus

^Δ^: *p *< 0.01 compared with control group

### Expression change of c-fos oncogene mRNA

**Figure 3:**
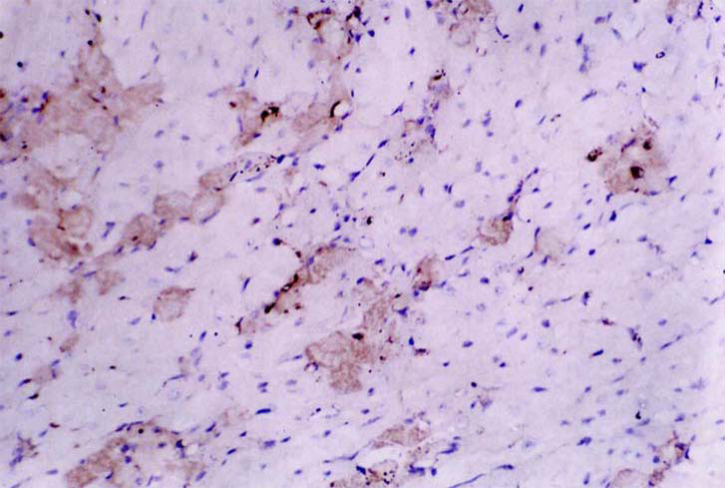
**The expression of c-fos mRNA in cardiomyocytes of VMC mice at 7 days after virus inoculation (200×)**.

A few c-fos mRNA expression positive cardiomyocytes were observed in mice of the control group. Positive c-fos mRNA expression in cardiomyocytes increased at 3 and 7 days after virus inoculation (Table 2, Figure 3).

**Table 2:** The expression change of c-fos oncogene mRNA in VMC mice

Group	Number	NPC/HPF	NPC/NTC(%)
D_3_	7	28.22 ± 10.31^Δ^	6.79 ± 2.34^Δ^
D_7_	8	52.24 ± 16.69^Δ^	12.85 ± 4.73^Δ^
Control	10	6.76 ± 2.35	1.64 ± 0.56

NPC: number of positive cardiomyocyte; NTC: number of total cardiomyocyte

^Δ^: *p *< 0.01 compared with control group

### The results of Medicine treatment

**Figure 4:**
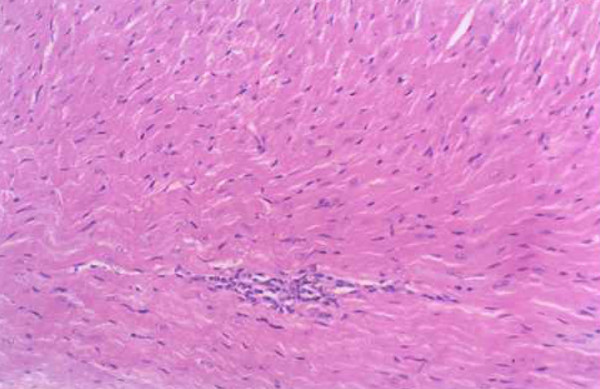
**Myocardial lesions in mice of the c-fos monoclonal antibody treatment group (200×)**.

**Figure 5:**
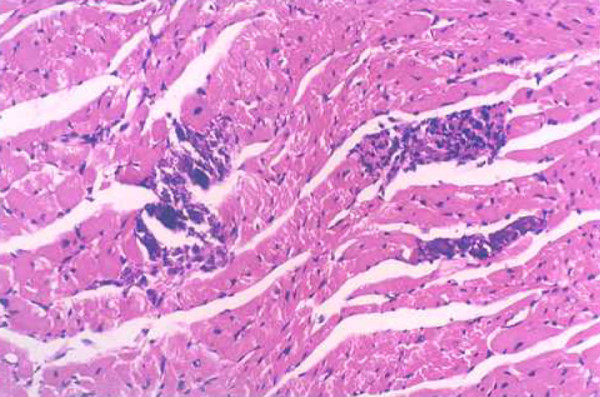
**Myocardial lesions in mice of the isoproterenol treatment group (200×)**.

**Figure 6:**
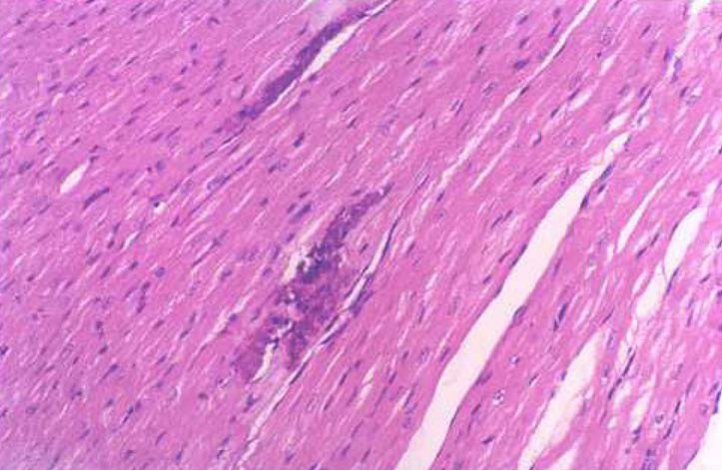
**Myocardial lesions in mice of the normal saline treatment group (200×)**.

Infiltration of the inflammatory cells and necrotic areas were decreased in the c-fos monoclonal antibody treatment group (Group E_1_) compared with the control normal saline treatment group (Group E_3_) at 7 and 15 days after virus inoculation, infiltration of the inflammatory cells and necrotic areas were increased in the isoproterenol treatment group (Group E_2_) (Table 3, Figures 4, 5 and 6).

**Table 3:** Effect of medicine treatment on myocardial lesions of VMC mice

Group		7d			15d	
	
	Number	I	N	Number	I	N
E_1_	13	1.21 ± 0.53^Δ^	0.97 ± 0.43*	12	0.94 ± 0.52*	0.72 ± 0.38*
E_2_	7	2.23 ± 0.91*	1.96 ± 0.79^Δ^	9	1.88 ± 0.81^Δ^	1.67 ± 0.70^Δ^
E_3_	8	1.85 ± 0.64	1.32 ± 0.55	10	1.31 ± 0.63	1.08 ± 0.52

I: Infiltration of the inflammatory cells; N: necrotic areas

*: *p *< 0.05 compared with Group E_3;_

^Δ^: *p *< 0.01 compared with Group E_3_

## Discussion

A broad range of extracellular signals trigger cells to adapt and grow according to their environment. Proto-oncogenes play an important role in signal transduction, a process that converts external stimuli into intracellular signals that guide cellular function. Within the past 10-15 years of oncogene research, the identification of proto-oncogenes as specific components of signal transduction pathways has been a major discovery in the field. These and other recent findings suggest that several new areas are emerging as important topics for future investigation in molecular oncogenesis.

Although the study of oncogenes has provided some useful insights into cancer mechanisms, the most beneficial aspect has been the delineation of the growth factor response pathway and molecular characterization of various important cellular processes. The nuclear proto-oncogenes c-fos and c-jun have been particularly useful in this regard. These studies have provided important information about gene regulation in response to growth factors, regulation of immediate early genes, and the function and interaction of transcription factors.

The Fos oncogene was discovered as the cellular homologue of three distinct tumor viruses derived from mice and chicken [2]. Both the normal and viral Fos transforming proteins complex with a 39-KD protein [3]. The genes c-fos and c-myc were the first to be identified as immediate early genes after detailed analysis of their mRNA expression patterns. The transient induction of c-fos expression is mediated by multiple transacting factors. The c-fos mRNA and its 55-kDa nuclear phosphoprotein (Fos) are rapidly, but transiently, induced by both growth factors and differentiating agents [4].

c-Fos may play a role as a potent inducer of apoptosis in pro-B cells and Ig class-switching B cells. c-Fos induced apoptosis is further supported by findings that induction of c-Fos expression is an early event in many cases of mammalian apoptosis [5,6]. Moreover, reduction of c-Fos activity by antisense oligonucleotides is able to prevent growth factor-deprived lymphoid cells from undergoing apoptosis. These results suggest that c-Fos may indeed have a protective function, including DNA repair, against harmful consequences of agents [7].

The proto-oncogene c-fos encodes a nuclear phosphoprotein (c-Fos). c-Fos, in a complex with products of another proto-oncogene, c-jun (AP-1), regulates the expression of AP-1 binding genes at the transcriptional level [8]. Overexpression of c-fos may play roles in some diseases, such as Alzheimer's disease, arthritis, myocardial stunning, neonatal hypoxia-ischemia, cardiac ischemia-reperfusion, and heart failure [9-18].

The c-fos protein expression induced by arthritis was found in rats, and pathological pain following arthritis activated pain sensitive neurons and evoked c-fos expression in spinal cord. Overexpression of c-Fos in the central nervous system is induced by some pathological stimulation. c-fos mRNA was overexpressed in the hippocampal neurons of the patients with Alzheimer's disease [9]. In rat models of myocardial stunning (MS), the expression of Fos protein increased apparently. Therefore, Fos may play a role in MS since it has close relation to injury repair of the molecule [10]. Cerebral hypoxia and/or ischemia also produce hyperexpression of specific genes(c-fos, c-jun) which may be involved in the mechanisms of excitotoxic neuronal death. Overall, Fos expression is mainly associated with cellular damage and subsequent death following hypoxic-ischemic injury.

Gonzalez CA et al. [11] assayed the Fos protein using immunohistochemical staining, and showed that the administration of naloxone methiodide or naloxone to morphine-dependent rats induced a marked Fos immunoreactivity within cardiomyocyte nuclei. Western blot analysis revealed a peak expression of c-fos in the right and left ventricles after naloxone methiodide withdrawal. Fos expression was increased after naloxone administration to morphine-dependent rats. These results suggested that the activation of c-fos expression observed during morphine withdrawal in the heart is due to intrinsic mechanisms outside of the central nervous system (CNS). To analyze differential gene expression after myocardial ischemia-reperfusion, Nelson assayed humans for the related immediate early genes c-fos and c-jun with *in situ *hybridization and also performed testing on lamb myocardium subjected to cardiopulmonary bypass with myocardial ischemia. The results showed that c-fos and c-jun were induced in ischemia-reperfusion myocardium at endcardiopulmonary bypass. Expression patterns of c-fos and c-jun by *in situ *hybridization were markedly different; myocardial c-fos expression was diffuse and homogeneous, whereas c-jun expression was patchy with areas of intense focal localization [12].

Since TNF-α and other cytokines increase apparently in VMC [19-21], Isoproterenol, TNF-α and other cytokines induce expression of c-fos and the c-jun oncogene [22-25], we deduced that abnormal expression of c-Fos can be observed in VMC. In our experiment, protein expression of c-Fos increased apparently compared with control mice at 3 days after virus inoculation, and increased further with the advance of disease. Expression peaked at 7-9 days, decreased gradually, and then became almost normal at 35 days after virus inoculation. The expression of c-fos mRNA in VMC mice was also higher than that of the control group at 3 days and 7 days after virus inoculation. Results indicated that the expression of c-fos increased in cardiomyocyte of VMC mice, and that c-Fos can compose AP-1 with c-jun gene products.

TNF-α also stimulated collagenase gene transcription. This stimulation is mediated by an element of the gene that is responsive to the transcription factor AP-1, and then the product of collagenase increase. Collagenase plays an important role in the course of tissue inflammation [26,27]. Therefore, we deduced that abnormal expression of c-fos may play a role in an inflammatory disease, specifically, VMC. In addition, c-fos can also regulate the transcription of apoptosis-related genes and thereby regulate cardiomyocyte apoptosis indirectly [28] in VMC. In our experiment, infiltration of the inflammatory cells and necrotic areas were decreased after c-fos was neutralized by c-fos monoclonal antibody treatment compared with the control normal saline treatment group. Infiltration of the inflammatory cells and necrotic areas were increased after increase of c-fos due to stimulation by isoproterenol. Our results show that c-fos plays an important role in myocardial lesions and is likely to be involved in the pathogenesis of VMC.

## Conclusions

c-fos expression in the cardiomyocytes of VMC mice is significantly increased, c-fos plays an important role in myocardial lesions. The apparent increase in expression of c-fos is likely to be involved in the pathogenesis of VMC.

## List of abbreviations

VMC: Viral myocarditis; SABC: Streptavidin biotin-peroxidase complex; CVB_3_: Coxsackie virus B_3_; PBS: Phosphate-buffered saline; SD: Standard deviation; MS: Myocardial stunning;

## Competing interests

The authors declare that they have no competing interests.

## Authors' contributions

SZ participated in the conception and design, acquisition of data, analysis and interpretation of data, drafted the manuscript, and performed the statistical analysis, carried out the immunohistochemical analysis and in situ hybridization. BH participated in the conception and design, acquisition of data, analysis and interpretation of data, drafted the manuscript, carried out the immunohistochemical analysis and in situ hybridization, and establishing of animal model. SG participated in the conception and design, interpretation of data, and carried out specimen collection. JG carried out the acquisition of data, analysis and interpretation of data, and drafted the manuscript, carried out the establishing of animal model, specimen collection. ZW carried out the acquisition of data, analysis and interpretation of data, and drafted the manuscript. GR participated in the establishing of animal model, specimen collection. All authors read and approved the final manuscript.
